# Frameless Single Robotic Radiosurgery for Pulmonary Metastases in Colorectal Cancer Patients

**DOI:** 10.7759/cureus.7305

**Published:** 2020-03-18

**Authors:** Jobst C Von Einem, Sebastian Stintzing, Dominik P Modest, Michael Wiedemann, Christoph Fürweger, Alexander Muacevic

**Affiliations:** 1 Hematology, Oncology, and Tumor Immunology (Campus Charité Mitte), Charité - Universitätsmedizin Berlin, Berlin, DEU; 2 Oncology, Charité - Universitätsmedizin Berlin, Berlin, DEU; 3 Radiation Oncology, European CyberKnife Center, Munich, DEU; 4 Medical Physics, European CyberKnife Center, Munich, DEU; 5 Stereotaxy and Neurosurgery, University Hospital, Cologne, DEU; 6 Radiosurgery, European CyberKnife Center, Munich, DEU

**Keywords:** colorectal cancer, pulmonary metastases, radiation, radiosurgery, cyberknife

## Abstract

Background

Surgical intervention and radiation therapy are common approaches for pulmonary metastasectomy. The role of minimally invasive techniques in pulmonary metastases remains unclear. Frameless single robotic radiosurgery [CyberKnife (CK); Accuray Incorporated, Sunnyvale, CA] of pulmonary metastases in colorectal cancer (CRC) patients offers high precision local radiation therapy.

Methods

We analyzed the efficacy and safety of CK treatment for lung metastases in CRC in 34 patients and a total of 45 lesions. The primary endpoint was local control (LC); secondary endpoints were progression-free survival (PFS), overall survival (OS), distant control (DC), and safety-relevant events.

Results

Of the treated lesions, 34/45 (77.8%) decreased in size or remained unchanged [complete response (CR), partial response (PR), stable disease (SD)]; 8/45 (17.8%) lesions increased in size [progressive disease (PD)] and 2/45 (4.4%) lesions were not evaluable. Local progression was shown in 2 lesions (4.4 %). The median PFS period was six months. In a median follow-up time of 19.4 months, medium OS was 19.9 months (range: 3-61 months). Distant recurrence was observed in 21/34 patients (61.8 %). Intrapulmonary progression occurred in six patients. In 4/45 cases, fiducial placement led to a pneumothorax; three out of four patients needed chest tube insertion. No radiation-associated side effects were reported in 57.8% of patients. In 10/45 cases (22.2%), patients suffered asymptomatic radiographic changes; 7/45 cases (15.6%) reported a late onset of radiation-associated side effects. Maximal radiation-associated side effects reached the Radiation Therapy Oncology Group/European Organization for Research and Treatment of Cancer (RTOG/EORTC) Grade 1.

Conclusion

CK treatment of pulmonary metastases is safe and well-tolerated. For metastatic colorectal cancer (mCRC) patients with pulmonary metastases and not eligible for surgery, CK radiation offers a valuable treatment option.

## Introduction

About 50-60% of all patients with colorectal cancer (CRC) develop metastatic disease [metastatic colorectal cancer (mCRC)] [1]. The liver and lung are the most frequent metastases sites of CRC [2]. Approximately 20% of all patients suffer from pulmonary metastases [3,4]. In 8-10% of CRC patients, pulmonary metastases occur after previous liver metastases [5]. The development of novel therapeutic regimens, agents, and combinations have led to higher response rates and longer overall survival [6-8]. After complete pulmonary metastasectomy, 5-year survival rates of up to 40% after resection have been reported [9,10]. Since not all patients with pulmonary metastases are eligible for surgical treatment and since techniques are heterogeneous, new treatment options are needed [1,5,11].

Surgery is the gold standard of care for metastasectomy. Some well-established methods in the treatment of liver metastases have been summarized as a “toolbox” to alternatively treat liver metastases if surgery is technically not possible or refused by the patient [12]. For lung metastases, these “tools” are limited or questionable with regard to efficacy and toxicity; for instance, radiofrequency ablation (RFA) of peripheral pulmonary tumors have shown heterogeneous results and side-effects [13,14].

It has been reported that non-invasive stereotactic body radiotherapy (SBRT) can reach 2-year local control (LC) rates of approximately 90% in patients electing not to undergo surgery [14]. This technique usually uses high, hypofractionated dosing and treats small volumes with tight margins [14]. The development of precise tracking options, such as fiducial placement, spine- or lung-tracking, allows the application of eradicating doses to pulmonary metastases and simultaneously spares the healthy lung tissue [15,16].

Encouraged by several reports on radiosurgery treatment in liver metastases and promising reports on radiosurgery treatment in lung metastases, we retrospectively analyzed 34 patients with a total of 45 lesions receiving single-session frameless robotic radiosurgery for CRC with lung metastases [1,17,18,19].

## Materials and methods

Study design

The conducted investigation is a retrospective analysis of the efficacy and safety of single-session robotic radiotherapy of pulmonary metastases in patients suffering from mCRC. The primary endpoint was LC; secondary endpoints were progression-free survival (PFS), overall survival (OS), and distant control (DC).

Patients

Patients who underwent the procedure from June 2008 to April 2013 were included in our study. Prior to CyberKnife (CK; Accuray Incorporated, Sunnyvale, CA) treatment, patients were reviewed by the multidisciplinary thoracic tumor board of the University Hospital Munich, Grosshadern. Patients suffered from surgically non-resectable pulmonary metastases of CRC. Some patients were also suffering from non-pulmonary metastases as well. Furthermore, patients not willing to undergo surgery in the first place were treated. Pre-treatment included chemotherapy in accordance with the local guidelines, radiotherapy, and surgical metastasectomy. The maximum diameter of metastases had to be less than 5 cm, which was to be determined by a CT scan before the treatment session. Additionally, the total irradiated volume was not to exceed 95 ccm. Patients had a lung function test before the intervention. Prior to the first treatment, informed consent to data evaluation was given by all patients in accordance with the guidelines of the local research ethics committee.

Evaluation of response to treatment

To evaluate the response of irradiated pulmonary metastases, the patients underwent CT scans in an interval of 8-12 weeks. Radiological response evaluation criteria in solid tumors (RECIST) guidelines were used. As differentiation between tumor re-growth and radiogenic inflammation in the RECIST were difficult to apply, short-time follow-up CT scans were performed if clinically indicated. LC was defined as tumor shrinkage or no tumor progress as evaluated in thoracic CT scans. An increase of the tumor volume over 25% compared to the pre-treatment dimension was defined as local recurrence. Besides, distant recurrence was defined as recurrence in the opposing pulmonary or extra-pulmonary sides.

Evaluation of toxicity

Side effects were classified as radiation-associated and not-radiation-associated. Pulmonary toxicity was evaluated using pulmonary function tests or CT scans. Furthermore, all patients underwent follow-up evaluations focusing on typical pulmonary symptoms. Morbidity due to marker placement (bleeding, pain) and morbidity due to radiation treatment were evaluated separately. Side effects due to radiation were graded according to the toxicity criteria of the Radiation Therapy Oncology Group (RTOG) and the European Organization for Research and Treatment of Cancer (EORTC). All other safety-relevant events were documented and evaluated.

Marker placement and tracking

Most patients underwent CT-guided percutaneous placement of one or two cylindrical gold fiducials (AB Medica, Milan, Italy), 5-mm long and 0.5 mm in diameter, directly into the metastases prior to radiation. This was done under local anesthesia. Patients had to sign an informed consent, which included warnings of following possible complications: pneumothorax, pulmonary hemorrhage, inadvertent placement of fiducial markers in the pleural space, fiducial marker migration, and unsatisfactory fiducial marker position for use during stereotactic radiotherapy. For patients refusing marker placement or for those not found eligible for marker placement, tracking was done by spine- or lung-tracking.

Radiation

The 3D target volume was identified on non-contrast-enhanced CT scans. The dose distribution was calculated using the Monte Carlo dose calculation. To cover microscopic tumor spread, a safety margin of 6 mm was added to the tumor diameter in all three dimensions. All lesions were treated with single-session radiosurgery to a maximum dose of 26 Gy. The respiratory motion of the lesion was tracked continuously by a 6-MV compact linear accelerator (LINAC), which was mounted on a six-axis robotic manipulator (CK). Based on the correlation between infrared markers on the patient´s chest tracked continuously with external cameras and the position of fiducials detected by two orthogonally positioned X-ray detectors, the position of the linear accelerator was corrected in real time. The radiation beam was directed from a multitude of angles around the patient. After this procedure, which lasted 45 minutes on average, patients were discharged from the institute immediately.

Statistics

Statistical analysis was done using STATA 10.1 for Macintosh (StataCorp, College Station, TX), IBM SPSS Statistics version 21 (IBM, Armonk, NY), and SAS 9.2 (SAS Institute Inc., Cary, NC).

## Results

There was a total of 45 metastases in 34 CRC patients [male 25 (55.6%), female 20 (44.4%)]. The median age was 65 years (range: 41-77 years). Reasons for surgical non-resectability were heterogeneous and included a high number of concomitant diagnoses, prior surgery, or difficult localization of the metastases. The patient characteristics are detailed in Table 1.

**Table 1:** Patients characteristics C: central; P: peripheral; KPS: Karnofsky Performance Status; UL: upper lobe; LL: lower lobe; ML: middle lobe; N.N: nomen nescio (not known); Rtx: radiotherapy

Patient	Age, years	Sex	KPS, %	Localization (C/P)	Localization (UL/ML/LL/N.N.)	Irradiated volume, ccm	Beams	Total dose, Gy	Target cover, %	Prior chemotherapy	Prior surgery	Prior radiotherapy	Prior local therapy	Follow-up (months)	Death
1	68	M	100	P	UL	20.9	106	22	99.7	Yes	No	No	None	43	Yes
				P	UL	25.0	117	24	96.58	Yes	No	No	None	31	Yes
2	59	M	100	P	LL	15.2	42	22		Yes	No	Yes	Rtx	6	Yes
				P	LL	11.2	137	22	99.81	Yes	No	Yes	Rtx	6	Yes
3	66	F	100	P	LL	17.9	133	22	97.5	Yes	Yes	Yes	Surgery + Rtx	27	N.N.
4	59	M	100	P	LL	15.4	54	22	99.07	Yes	No	No	None	50	Yes
				P	LL	21.2	138	24	96.13	Yes	No	Yes	CyberKnife	41	Yes
5	43	F	100	C	UL	2.3	68	22	89.61	Yes	Yes	No	Surgery	61	No
				P	UL	5.3	42	26	96.07	Yes	Yes	Yes	Surgery + Rtx	18	No
6	52	F	100	P	LL	5.0	62	24	99.17	Yes	No	No	None	48	No
7	64	F	100	P	ML	7.9	116	24	99.51	Yes	Yes	No	Surgery	54	No
				P	UL	5.9	123	26	100	Yes	Yes	Yes	Surgery + Rtx	39	No
				P	ML	33.5	226	26	98.01	Yes	Yes	Yes	Surgery + Rtx	39	No
8	70	M	100	P	LL	18.1	144	22	98.48	Yes	Yes	No	Surgery	31	Yes
9	56	M	100	P	UL	17.3	108	24	97.37	Yes	Yes	No	Surgery	44	No
10	70	M	100	P	LL	21.1	159	26	97.38	Yes	No	No	None	8	No
11	55	F	100	C	LL	3.4	33	20	99.71	Yes	Yes	No	Surgery	23	Yes
12	73	M	100	C	UL	9.1	117	24	80.2	Yes	No	No	None	31	Yes
13	76	M	100	C	LL	14.9	102	26	98.87	Yes	Yes	No	Surgery	35	No
14	50	F	100	P	UL	7.2	52	26	96.84	Yes	No	No	None	32	No
15	47	M	100	P	UL	4.6	47	26	96.4	Yes	Yes	No	Surgery	9	No
16	72	F	100	C	UL	71.6	199	22		Yes	Yes	No	Surgery	13	No
17	58	F	100	P	LL	24.7	168	26	98.74	Yes	Yes	No	Surgery	19	Yes
18	66	M	100	P	N.N.	25.1	56	26	99.39	Yes	Yes	Yes	Surgery + Rtx	14	Yes
19	56	F	100	P	LL	7.7	106	26	99.59	Yes	Yes	No	Surgery	17	No
20	66	M	100	C	UL	90.4	151	19	99.68	No	Yes	Yes	Surgery + Rtx	4	Yes
21	66	F	90	C	UL	21.7	178	26	95.53	Yes	Yes	No	Surgery	17	Yes
22	63	M	100	P	LL	5.0	56	26	93.32	Yes	No	No	None	2	No
23	67	M	100	P	UL	25.8	49	26		No	Yes	No	Surgery	0	N.N.
24	72	F	100	P	LL	13.2	43	26	99.51	No	No	Yes	Rtx	13	No
25	66	M	100	P	LL	9.0	48	26	98.9	Yes	Yes	No	Surgery	17	No
				C	LL	19.8	156	26	98.09	Yes	Yes	Yes	Surgery + Rtx	13	No
				P	ML	21.5	109	26	97.64	Yes	Yes	yes	Surgery + Rtx	4	No
26	55	M	100	P	LL	23.3	110	26		Yes	No	No	None	4	N.N.
27	41	F	100	C	UL	6.0	48	26		Yes	No	No	None	7	No
				P	LL	10.0	219	26	98.61	Yes	No	Yes	Rtx	6	No
28	77	F	90	C	LL	68.1	228	45	98.19	Yes	Yes	Yes	Surgery + Rtx	10	Yes
29	72	M	90	C	LL	24.3	65	26	91.4	Yes	No	No	None	9	No
				C	LL	29.0	147	26	98.92	Yes	No	Yes	Rtx	9	No
30	74	M	100	P	LL	24.7	99	26		Yes	Yes	No	Surgery	4	N.N.
				P	UL	6.2	175	26	91.86	Yes	Yes	Yes	Surgery + Rtx	2	N.N.
31	48	F	90	P	UL	5.1	49	26	92.92	Yes	Yes	No	Surgery	3	No
32	75	M	100	P	LL	22.7	157	26	86.56	Yes	No	No	None	2	No
33	43	F	100	P	LL	9.4	116	26	98.46	Yes	No	No	None	4	No
34	53	F	100	C	LL	5.2	65	26	96.7	Yes	No	No	None	5	No

**Figure 1:**
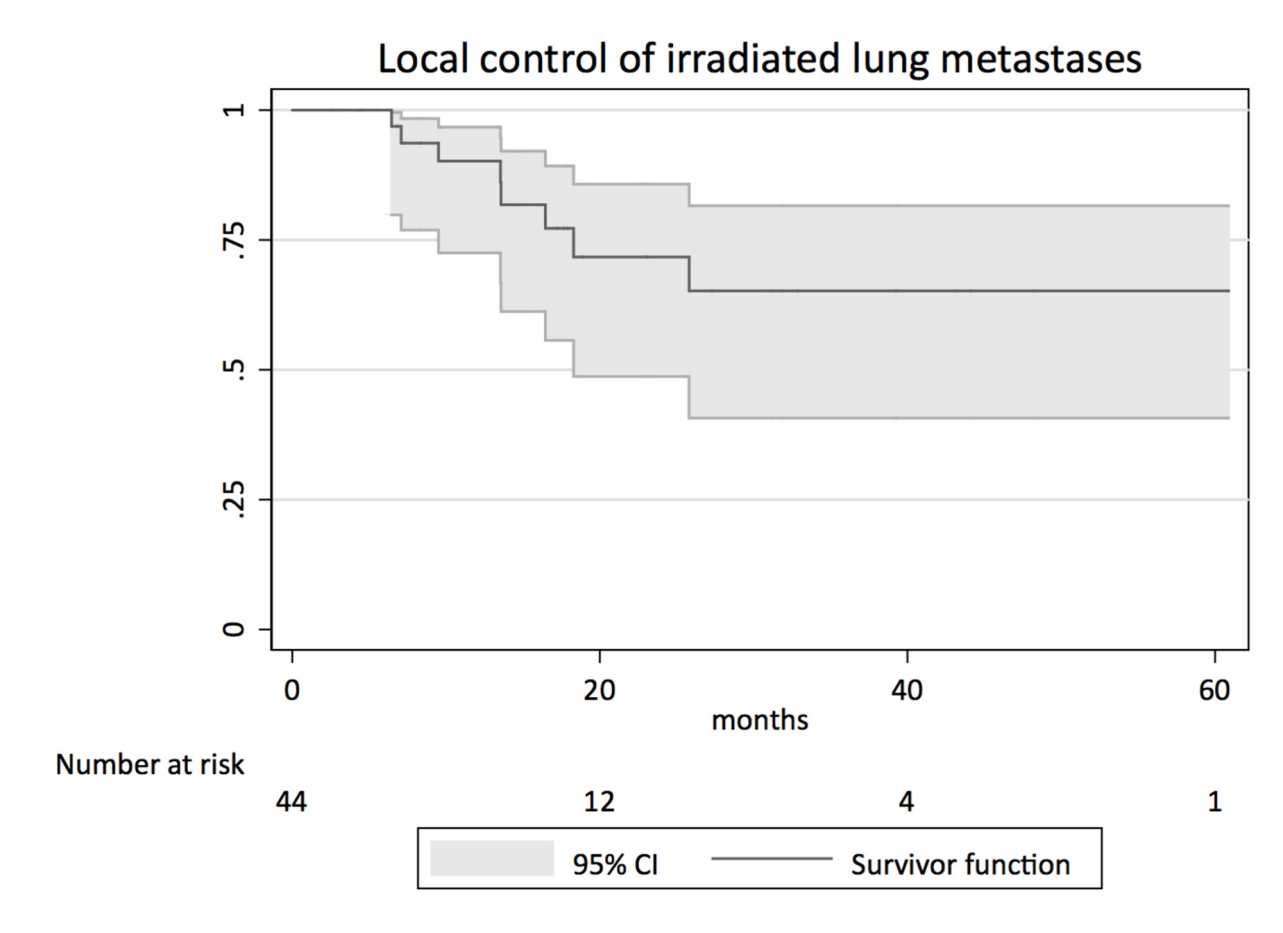
Kaplan-Meier survival analysis for local control of irradiated lung metastases CI: confidence interval

In terms of LC, 35/45 treated lesions (77.8%) decreased in size or remained unchanged [complete response (CR), partial response (PR), stable disease (SD)], 8/45 (17.8%) lesions increased in size [progressive disease (PD)], and 2/45 (4.4%) lesions were not evaluable due to missing follow-up scans. Local progression within the previously irradiated lesion was shown in two lesions (4.4 %) (Figure 1). 

**Figure 2:**
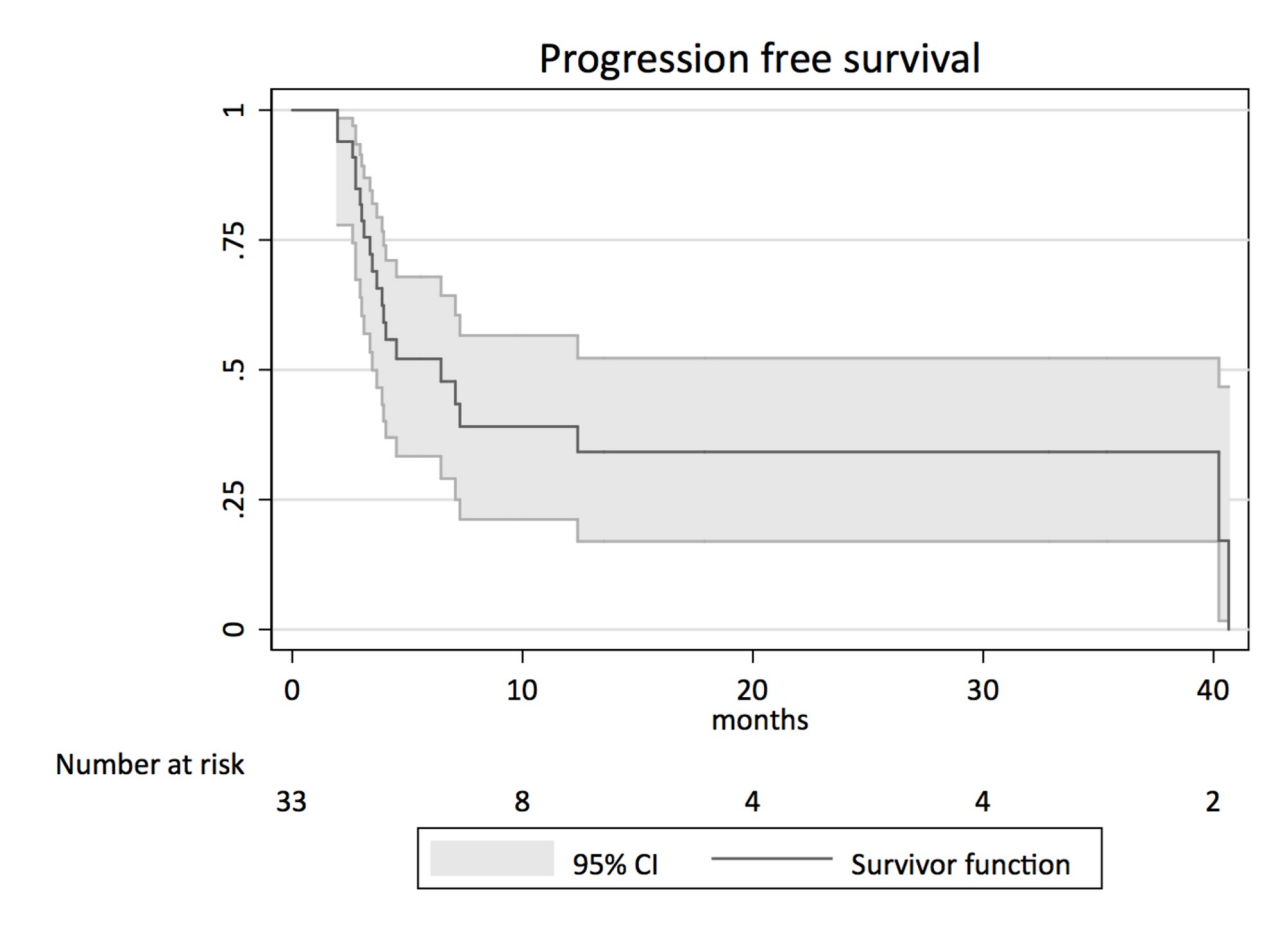
Kaplan-Meier survival analysis for progression-free survival CI: confidence interval

Median PFS, calculated with the Kaplan-Meier method, was six months. (Figure 2).

**Figure 3:**
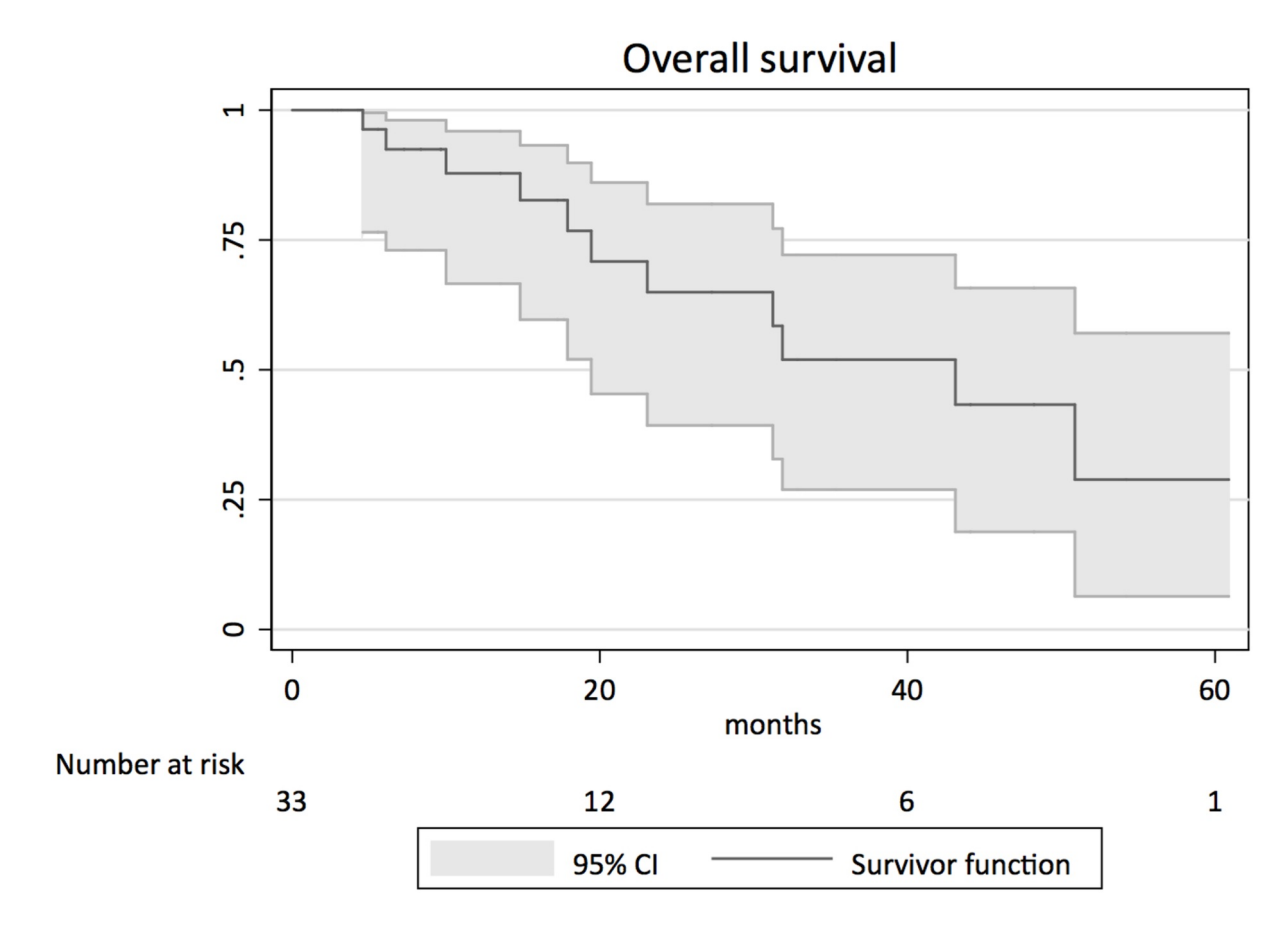
Kaplan-Meier survival analysis for overall survival CI: confidence interval

Within a median follow-up time of 19.4 months, medium OS was 19.9 months (range: 3-61 months) (Figure 3).

Distant recurrence outside the irradiated lung was observed in 21/34 patients, representing 61.8 % of all patients. Intrapulmonary progression in previously non-irradiated sections occurred in six patients.

In 4/45 cases, fiducial placement led to a pneumothorax; three out of these four patients needed chest tube insertion afterward. No radiation-associated side effects were reported in 57.8% of patients. In 10/45 cases (22.2%), patients suffered from acute radiation-associated side effects; 7/45 cases (15.6%) reported a late-onset of radiation-associated side effects. According to the toxicity criteria, RTOG/EORTC maximal radiation-associated side effects reached Grade 1 and were described as asymptomatic pneumonitis.

With a median number of 109.4 beams, a median target cover of 96.7%, and a median isodose of 65.9%, tumors with a mean planning target volume of 18.9 ccm were treated with a median dose of 25.2 Gy.

## Discussion

The approach towards oligometastatic disease in mCRC patients not suitable for or rejecting surgery is challenging, and no standard treatment has been defined so far [12]. The current European Society for Medical Oncology (ESMO) guidelines for the management of metastatic colorectal cancer, therefore, define the local ablative treatment options as “toolbox” [20]. Still, there are notable differences in invasiveness and side effects that require further investigations and ask for the proof of efficacy as compared to surgery, which remains the standard local ablative treatment method. The use of radiation treatment, including SBRT, has shown promising results but data from randomized trials comparing the different local ablative techniques to each other or to surgery is still scarce [12,21].

We report the results of a homogeneously treated group of 34 patients (and a total of 45 lesions) with 1-3 pulmonary metastases due to CRC treated with SBRT using the CK system focusing on safety and efficacy. In terms of safety, we did not determine any radiation-induced toxicity higher than grade 1 pneumonitis (10 patients), defined as asymptomatic radiographic changes. Our findings stand as a testament to the low toxicity and favorable treatment-associated morbidity of single-fraction radiosurgery in patients with pulmonary metastases due to CRC. None of those patients suffered from chronic side effects. In particular, no skin reactions, necrosis, or any other treatment-related toxicities were observed.

Similar treatment was performed by Hof et al. who treated 61 patients with 71 pulmonary metastases of different origins (8/61 suffered from pulmonary metastases due to mCRC) with stereotactic single-fraction treatment [22]. In this cohort, three patients developed grade 3 toxicity requiring treatment and oxygen for pneumonitis. No grade 4 toxicities occurred. The majority of the patients had grade 1 and 2 toxicities. In their cohort, 70.4% of all patients developed perifocal changes of the normal lung tissue, detectable by CT scans. Hof et al. displayed no statistically significant correlation between the occurrences of perifocal changes and the tumor volume and the administered radiation dose [22]. But due to the different tumor types treated and missing subgroup analysis, the efficacy of SBRT for CRC lung metastases remained unclear.

Another study by Rusthoven et al. included 68 lesions in 38 patients in a multi-institutional phase I/II clinical trial in which they received SBRT in three fractions with doses up to 60 Gy (9/38 suffering from pulmonary metastases due to mCRC). These patients showed no grade 4-5 toxicities. But three patients experienced grade 3 toxicity, including pneumonitis with increased dyspnea, oxygen requirement and a decrease in FeV1, rib fracture after SBRT, and confluent moist desquamation of the skin. Furthermore, four patients developed grade 2 radiation dermatitis, which resolved within three months [18].

The safety of the gold standard procedure, the surgical pulmonary metastasectomy, was investigated in 171 mCRC patients by Meimarakis et al. [3]. Of their 238 surgical interventions (n = 171 patients), 156 were performed as wedge resections, 30 as segmental, and 52 as major resections (35 lobectomies, 4 bilobectomies, 1 sleeve bilobectomy, and 12 pneumonectomies). Complications developed in 10 patients. Acute rethoracotomy was performed in one patient with hemorrhagic pleural effusion and empyema. Four patients developed severe pneumonia. Two patients died postoperatively (1.2%) [3]. In another study, it was shown that peri- and postoperative complications numbers were comparable to the data presented by Meimarakis et al. and included re-intubation, pleural empyema, partial atelectasis, pulmonary embolism, and superficial wound infections [4]. In our cohort, four cases of fiducial placement led to a pneumothorax; three of these four patients needed chest tube insertion. As new techniques such as MR-guided radiotherapy arise, this might be discussed differently; but for now, this approach can be considered safe. In terms of efficacy, an LC rate of 77.8% (35/45 patients), a median PFS of six months, and a median OS of 19.9 months have been achieved in our study. This is comparable to other non-surgical therapy approaches, but data varies among different local treatment options.

Success in terms of surgery is defined by completeness and margin. LC in resected patients with tumor negative margins is higher and OS, ranging from 25.2 to 51 months, is longer when compared to patients in our study [3,4,23]. In these surgery based trials, survival rates differed according to the completeness of pulmonary metastasectomy [3,4]. Meimarakis et al. have stated that the surgical resection of metastases improves survival significantly and coherently that an incomplete resection is associated with poorer outcomes when compared to chemotherapy alone in patients with hepatic and pulmonary colorectal metastases [4]. They showed that complete resection of the pulmonary and hepatic metastases led to a median survival of 66.8 months (31.5 months in patients with incomplete resection) and 30.1 months in patients treated with chemotherapy alone. In their isolated lung metastases cohort, the median survival rate was 35.2 months [3]. 1-, 3-, and 5-year survival rates for patients following R0 resection were 88.8, 52.1, and 32.9 % respectively. Almost identical results were presented by Schuele et al. whose cohort of isolated pulmonary metastases of colorectal primary reached a median survival rate of 33.3 months after surgical metastasectomy [23].

All surgical studies are troubled by the unresolvable bias that holds that only patients with an adequate pulmonary function are suitable for surgical procedures. As an alternative, and for surgically inoperable patients with limited pulmonary metastases, radiation therapy and SBRT in particular may be an option as OS rates for these procedures at 24 months are 33% and 86% and LC rates are 53% to 100% respectively [19,21,24-28].

A retrospective comparison of outcome after conventional, multi-session SBRT for pulmonary metastasectomy in patients with pulmonary oligometastasis was performed by Widder et al. [26]. In their study, pulmonary metastasectomy was offered as the first-choice treatment and conventional SBRT was offered for patients they considered to be less suitable surgical candidates. More unfavorable prognostic factors were present among the patients treated with conventional SBRT: they were significantly older, had a different distribution of primary tumor origins, and had a shorter metastasis-free interval. In a median follow-up time of 43 months, estimated OS rates at one, three, and five years were 87%, 62%, and 41% for metastasectomy, and 98%, 60%, and 49% for SBRT, respectively. Therefore, and despite the above-mentioned selection bias, survival after SBRT was comparable to pulmonary metastasectomy [26].

In terms of technique and in contrast to surgery as well as conventional SBRT, patients in our study presented only once for therapy procedures. In these end-stage tumor patients, time outside a medical institution may be a reasonable marker for the quality of life and thus favors time-efficient procedures.

Our data is limited by several factors. The study was performed retrospectively and at a single center. Even though our cohort of 34 patients (with a total of 45 treated lesions) is comparable to most other studies in this field, the population is still rather small. Furthermore, most of the patients suffered from the oligometastatic disease. Pretreatment conditions, performance status, age, sex, and tumor locations differed partly significantly. Therefore, it is difficult to refer to the OS outcomes of radiosurgery for pulmonary metastases exclusively.

## Conclusions

We can conclude that single-session frameless robotic radiosurgery is a safe, efficient, and convenient method to treat pulmonary oligometastases of CRC in patients not eligible for or rejecting surgery. Radiosurgery offers a treatment option with limited side effects and proficient LC. In accordance with the ESMO guidelines, SBRT using the CK device is an effective method in the "toolbox" to treat mCRC. Further prospective randomized studies are warranted to confirm the effectiveness of radiosurgery and to define the optimal fractionation and dosage.
